# Mr. Webster on Ann Moore's Case

**Published:** 1813-08

**Authors:** John Webster

**Affiliations:** Member of the Royal College of Surgeons, London. Derby


Member of the Royal College of Surgeons, London.

*To the Editors of the Medical and Physical Journal.*

GENTLEMEN,

MR. GRANGER having thought proper to forestal the Rev. Leigh Richmond's intended statement of the second watch and subsequent exposure of the impostor Ann Moore, I trust I shall be allowed to offer a few brief remarks remarks upon his paper which appeared in the last number of the Edinburgh Medical and Surgical Journal, called an Account of the second Watch at Tutbury, in which he must permit me to say he is not quite correct, and the errors appear to me to be material. The coming publication of the Rev. Leigh Richmond may, in the opinion of some persons, render any comment unnecessary; but one or two errors being of a nature not to be noticed by that gentleman, I hope I shall not be thought intruding upon the time of your readers in bringing the subject before them once more.

Mr. Granger says, "that on the tenth day the debility was so extreme as to occasion syncope, the pulse 140 and indistinct;" then in a note he says, "that it is now admitted that the illness was simulated for the purpose of exciting alarm." Now I would ask whether it was possible to feign (I do not like the word *simulate)* syncope with a pulse at 140 and indistinct? Where did he learn that delirium is always a precursory symptom of death from abstinence? Again, he says, "that on the ninth day a consultation of several gentlemen of the faculty was obtained, whose opinion was that Ann Moore was *moribund!*" Why use this obsolete term? The fact is, she never was in a state of syncope. On the ninth day she was evidently sinking from inanition, and the committee being alarmed, sent for Dr. Garlike that evening to visit her as soon as possible. He went over the next morning early, and found her exhibiting the usual appearances of a person in a dying state. He recommended the watch should instantly cease, and that the daughter should be immediately sent for, which was agreed to by the committee; and the presumption is, that being left to themselves the daughter administered some kind of nutriment to her, since in a few hours she began to revive, and the next day was nearly as well as usual. There is no proof that she swallowed any vinegar and water on the ninth day, and it is fair to presume she did not, for a spoonful of warm water was put into her mouth on the morning of the tenth, just before the watch ceased, by desire of Dr. Garlike, none of which she swallowed. Mr. Granger says she swallowed two spoonsful of milk, but does not say when, and therefore this information cannot be interesting to any one: it might be two days or a week after the watch ceased, and no one doubted that as soon as the daughter was admitted she took nourishment.

In page 79 of your last Number of the Medical and Physical Journal, you say that the observations of Dr. Henderson have been the means of bringing out a complete detection of the imposture practised by Ann Moore. Now this is not correct; correct:—in November last, previous to the appearance of Dr. Henderson's observations, it was proposed by the Rev. Leigh Richmond that she should undergo a second watch, which she consented to, and steps were then taken, with the concurrence and advice of other gentlemen, to carry it into execution. With the sequel you and the public are acquainted. This may appear trivial, but I think it right to correct a material error, and to show that Dr. Henderson's pamphlet had not any thing further to do with the second watch and subsequent exposure than perhaps somewhat accelerating it.

It seems to have been admitted by the most sceptical as to the stories of her living without food, that, as Mr. Granger says in the Edinburgh Journal, her lower extremities are in a state of paralysis. I have, however, the authority of Dr. Garlike (whose opinion agrees with my own) for stating that in his opinion her extremities never were paralytic, and that she has sensation and can move them. This opinion will probably appear in the Rev. Leigh Richmond's pamphlet. I am, Gentlemen,

Your obedient Servant,

JOHN WEBSTER,

Member of the Royal College of Surgeons, London.

*Derby, July* 7, 1813.

